# Maternal mental health during the COVID-19 lockdown in China, Italy, and the Netherlands: a cross-validation study

**DOI:** 10.1017/S0033291720005504

**Published:** 2021-01-13

**Authors:** Jing Guo, Pietro De Carli, Paul Lodder, Marian J. Bakermans-Kranenburg, Madelon M. E. Riem

**Affiliations:** 1 Department of Health Policy and Management, School of Public Health, Peking University, Beijing, China; 2 Department of Developmental and Social Psychology, University of Padua, Padua, Italy; 3 Department of Medical and Clinical Psychology, Center of Research on Psychological & Somatic Disorders, Tilburg University, Tilburg, The Netherlands; 4 Clinical Child & Family Studies, Faculty of Behavioral and Movement Sciences, Vrije Universiteit, Amsterdam, The Netherlands; 5 Behavioural Science Institute, Radboud University, Nijmegen, The Netherlands

**Keywords:** COVID-19 pandemic, cross-validation model, grandparent support, maternal mental health

## Abstract

**Background:**

The coronavirus disease 2019 (COVID-19) pandemic had brought negative consequences and new stressors to mothers. The current study aims to compare factors predicting maternal mental health during the COVID-19 lockdown in China, Italy, and the Netherlands.

**Methods:**

The sample consisted of 900 Dutch, 641 Italian, and 922 Chinese mothers (age *M* = 36.74, s.d. = 5.58) who completed an online questionnaire during the lockdown. Ten-fold cross-validation models were applied to explore the predictive performance of related factors for maternal mental health, and also to test similarities and differences between the countries.

**Results:**

COVID-19-related stress and family conflict are risk factors and resilience is a protective factor in association with maternal mental health in each country. Despite these shared factors, unique best models were identified for each of the three countries. In Italy, maternal age and poor physical health were related to more mental health symptoms, while in the Netherlands maternal high education and unemployment were associated with mental health symptoms. In China, having more than one child, being married, and grandparental support for mothers were important protective factors lowering the risk for mental health symptoms. Moreover, high SES (mother's high education, high family income) and poor physical health were found to relate to high levels of mental health symptoms among Chinese mothers.

**Conclusions:**

These findings are important for the identification of at-risk mothers and the development of mental health promotion programs during COVID-19 and future pandemics.

## Introduction

The coronavirus disease 2019 (COVID-19) pandemic presents the largest public health threat in at least a century (Hermann, Fitelson, & Bergink, 2020). To stop the community spread of COVID-19, almost all the countries implemented a range of public health and social measures aimed at reducing social contact (World Health Organization, 2020). Though these actions were highly effective in controlling the COVID-19 pandemic (Flaxman et al., 2020; Li et al., 2020), they also brought negative consequences and multiple new stressors (Bonaccorsi et al., 2020; Fegert, Vitiello, Plener, & Clemens, 2020), including physical and psychological health risks, isolation and loneliness, the closures of many schools and businesses, economic vulnerability, and job losses. Although the pandemic is likely to affect the daily lives of everyone, some individuals may be more vulnerable to the adverse circumstances of the pandemic than others. For instance, mothers with young children may be more affected by the pandemic, because they are often the primary caregiver. Due to the closures of schools and day care centers, mothers suddenly needed to combine remote working with homeschooling their children, in the absence of support from family or friends (Zhao et al., 2020). Recent studies have identified a substantial increase in the likelihood of maternal depression and anxiety during the COVID-19 pandemic (Davenport, Meyer, Meah, Strynadka, & Khurana, 2020). Since maternal mental health constitutes a well-known predictor of child development and adaptation (Weissman et al., 2006), more effort in understanding which factors are associated with psychopathology during the current pandemic is warranted. Given the global impact on the pandemic, it is important to examine predictors of maternal psychopathology across countries. The current study therefore aims to compare factors associated with maternal mental health during the COVID-19 lockdown in China, Italy, and the Netherlands.

### COVID-19-related stress, resilience and maternal mental health

During the COVID-19 pandemic mothers experience increased levels of psychological distress (Van den Heuvel et al., in preparation), which has previously been shown to be attributable to two types of pandemic-related stress: stress associated with daily life changes (pandemic-related life stress) and stress related to work changes (Hamadani et al., 2020; Klaiber, Wen, DeLongis, & Sin, 2020). Pandemic-related life stress refers to concerns about food, health, baby care, social interactions, while pandemic-related work stress refers to concerns about increased responsibilities, unemployment, and financial difficulties. However, not all parents experiencing cumulative stressors from COVID-19 may be at risk of higher perceived stress or poor mental health, suggesting that protective factors such as resilience and support may mitigate the impact of COVID-19 on maternal stress. Resilience refers to ‘the interactive and dynamic process of adapting, managing, and negotiating adversity’ (Goodman, Saunders, & Wolff, 2020). As one of many possible outcomes to life challenges, mothers may also develop resilience, which could help them to positively adjust and adapt to the adversity. Prime et al., points out that resilience as individual resource and COVID-19-related stress as external risk factor interact in predicting maternal vulnerability (Prime, Wade, & Browne, 2020). Indeed, recent studies have found that younger mothers with more daily stress and low resilience had worse mental health outcomes (Barber & Kim, 2020; Bruine de Bruin, 2020; Farewell, Jewell, Walls, & Leiferman, 2020). Given that each risk and protective factor alone only has a small effect, analyses incorporating multiple factors of COVID-19-related stress and resilience are essential, as they have the potential to reveal the interplay of such factors.

It follows from the above that some mothers may be more vulnerable in the COVID-19 epidemic than others. One study found that mothers with young children were particularly vulnerable during the COVID pandemic (Pierce et al., 2020). The family economic stress model provides a framework to understand how the pandemic impacted maternal mental health in mothers with young children and low socioeconomic status (Keating, Conger, & Elder, 1995). In particular, mothers with children aged 0–5 with poor physical health and food insecurity were found to be at the greatest risk for poor mental health (Linares, Azuine, & Singh, 2020). Thus, the number of children, age of the child, and mother's personal and context characteristics should be taken into account as factors predicting maternal mental health. Specifically related to the COVID-19 crisis, we expect that mothers with more children, young children, and low socioeconomic status are particularly at risk for poor mental health.

### Kinship networks, family conflict, and maternal mental health

A growing body of research has linked father involvement with various maternal health outcomes (Allport et al., 2018; Maselko et al., 2019). For example, mothers who feel supported by their partners show better mental health during the five years after birth (Meadows, McLanahan, & Brooks-Gunn, 2007). However, the relation between partner support and maternal mental health depends on the quality of paternal involvement (Waller, 2012). Co-parental conflict, undermining parenting behavior, and discrepancies in childrearing beliefs have been associated with maternal psychological distress and depression (Feinberg, 2003; Solmeyer & Feinberg, 2011). In addition, in the context of the COVID-19 pandemic, many countries reported increasing marital conflict, which may reflect low support from father and is generally associated with poorer mental health (Prime et al., 2020). Moreover, father involvement varies across countries. In Italy, where gender equality is low (UNDP, 2010), fathers generally show lower involvement in child care compared to European countries with higher gender equality (Craig & Mullan, 2011), such as the Netherlands.

Notably, at the family level, more attention has been paid to the nuclear family system, and relatively little is known about the role of kin (e.g. grandparents, relatives, neighbors) network support in affecting mothers' mental health (Leon & Dickson, 2019). Since the outbreak of COVID-19, stay-at-home orders have caused millions of children to remain out of school or childcare, and thus support from outside the family unit has abruptly reduced. Grandparents were advised to keep distance and, as a result, parents suddenly had solely each other to rely on. However, extended families may benefit from the presence of co-residential grandparents who offer support in child care and may lower the caregiving burden for mothers. Up to now, findings on the relation between grandparental support and maternal mental health are mixed. On the one hand, grandparents are important sources of support for mothers, and can help them dealing with the stress in crises. On the other hand, conflict is more likely to happen when grandparents are involved in childcare, which not only affects intergenerational relationships but also has a negative impact on mother's psychological health (Leung & Lam, 2012). Here, we investigate both the role of grandparental care and fathers' involvement as protective factors, and family conflict as a risk factor associated with mothers' mental health.

### Maternal mental health in China, Italy, and the Netherlands

Identifying mothers at-risk for poor mental health is important in order to provide support, but it is unclear whether the constellation of factors predicting poor maternal health is similar across countries. Due to the typically cultural and institutional variations between East and West, large differences in maternal mental health may exist between China and Europe, and between European countries (Burri & Maercker, 2014; Hessami, Romanelli, Chiurazzi, & Cozzolino, 2020). For example, differences in gender equality may play a role in these between-country differences. Mothers who receive low support from fathers in countries with lower gender equality, such as Italy, may be differently impacted by the crisis compared to mothers living in countries such as China where child care is commonly shared to a high extent with other family members, such as fathers and grandparents. We therefore examined the patterns of associations among COVID-19-related stress, resilience, and maternal mental health across three countries: China, Italy, and the Netherlands.

These countries were chosen as regions of interest because they differ substantially in gender equality and the way mothers share child care with others.

More specifically, these three countries show cultural differences in family structure and role division between mothers and fathers. In China, the traditional notion that ‘the man goes out to work while the woman looks after the house’ still prevails in many parts of mainland China (Wang, Yu, Zhu, & Ji, 2019). Differences in father involvement also exist between Europe countries. For example, one study reported the highest father involvement in Sweden and the lowest in Italy (Ragni & De Stasio, 2020). Compared to the Netherlands, gender inequality is high in Italy, and the rate of female employment is amongst the lowest in Europe (Hu et al., 2019). Dutch fathers are more involved in child care and household activities than Italian fathers. In addition, there are differences in the family structure across China, Italy, and the Netherlands. Due to the impact of the one-child policy, most Chinese families have fewer children than families in other countries. Compared to European families, grandparental involvement in child care is much stronger in China and may buffer pandemic-related distress for Chinese mothers. The proportions of grandparents providing childcare to grandchildren differ considerably between China (58%) (Ko & Hank, 2014) and European countries (around 7–11%) (Glaser et al., 2018). Within European countries, Italian grandparents are more often involved in assisting parents and taking care of their grandchildren than Dutch grandparents, although support from grandparents was diminished during the lockdown in both countries due to social distancing. Besides, the impact of grandparental support on maternal mental health may depend on the age of the child. Compared to older children, rearing young children requires considerable social, financial, and health care resources (Mistry, Stevens, Sareen, De Vogli, & Halfon, 2007). Grandparental support may therefore be particularly important for mothers with younger children (Lee & Gardner, 2015).

Here, we examined key factors relating to maternal mental health across three countries, China, Italy, and the Netherlands, using a cross-validation modeling approach (de Rooij & Weeda, 2020). With more lockdowns yet to come, identifying factors predicting poor maternal mental health is important for identifying mothers in need for support during (future) pandemics.

### Aims and hypotheses

The objective of this study was to compare risk and protective factors associated with mental health among mothers with children aged 1−10 years during the COVID-19 outbreak in China, Italy, and the Netherlands. We hypothesized that pandemic-related stress and resilience are shared factors, respectively, increasing and decreasing the risk for poor maternal mental health. We further hypothesize that factors varying across cultures, such as grandparental support, father involvement, and family structure characteristics, may be differently associated with mental health across the three countries. Specifically, due to co-residential grandparents in China, grandparental support may have a most pronounced protective influence on mental health for Chinese mothers.

## Methods

### Participants, study design, and procedure

Data were collected during the first wave of the COVID-19 pandemic, when lockdowns resulted in closures of schools and daycare centers. More specifically, timeframes for data collection were: April 17–May 10 for the Netherlands, April 21–June 13 for Italy, and April 21–April 28 for China. This study focused on the parents aged 18 years or older with at least one child between 1 and 10 years old.

In the Netherlands and Italy, a snowball sampling strategy was used to recruit parents by social media advertisements (Facebook, LinkedIn, Twitter), schools and daycare centers in Northern Brabant (the Netherlands), and Lombardy (Italy). Parents from others regions in Italy and the Netherlands were also allowed to participate. Dutch parents were also recruited from the Dutch I&O research panel (https://www.ioresearch.nl). Italian and Dutch participants were asked to respond to an online survey (https://www.qualtrics.com), while a web-based platform (https://www.wjx.cn/app/survey.aspx) was used for Chinese participants. In China, parents took part in the survey mainly from contacting elementary schools in Henan, Hubei, and Shenzhen city.

A sample size of 400 participants per country was required to achieve sufficient power to detect moderately sized associations (power = 0.80, *r* = 0.20, *α* = 0.05) between maternal mental health and each independent variable. After excluding participants who did not meet the inclusion criteria (e.g. they had only children older than 10 years, *n* = 8 Dutch parents, *n* = 47 Chinese parents), 1156 Dutch parents, 674 Italian parents, and 1243 Chinese parents were concluded as final sample. For the aims of the current study, fathers were excluded, resulting in a sample of 900 Dutch, 641 Italian, and 922 Chinese mothers.

### Measurements

#### Maternal mental health

Mental health was accessed by the Brief Symptom Inventory 18 (BSI-18, omitting suicidality) measuring somatization (6 items), depression (5 items), and anxiety (6 items), and a subset of 10 questions of the posttraumatic stress disorder (PTSD) checklist for DSM-5. All the questions apply to the two preceding weeks and were rated as ‘1 = never’, ‘2 = occasionally’, ‘3 = sometimes’, ‘4 = often’, ‘5 = Very often’. Since the four dimensions of mental health symptoms were highly correlated (0.776 < *r* < 0.961), total mental health scores were computed by averaging all 27 item scores. Confirmatory factor analysis supported this decision by indicating that one general psychopathology factor explained the correlational structure of the four latent psychopathology factors (RMSEA = 0.06; CFI = 0.974; SRMR = 0.043). The Cronbach's alpha estimate of reliability was 0.96.

#### Grandparental support

Parents were asked to indicate whether or not parents received support in child care from grandparents.

#### Father involvement

Father involvement was assessed via a self-designed questionnaire by asking the degree of fathers' contributions to 20 household chores or child care activities, such as homeschooling, bringing the child to bed, or washing clothes and so on. Mothers were asked to rate their own contribution and their partner's contribution to these tasks in the past week on a scale ranging from 1 (almost exclusively mother) to 5 (almost exclusively father). Mean scores were calculated, with higher scores representing greater involvement of father. The average of these 20 item scores was used as a measure of father involvement. Cronbach's alpha estimate of reliability was 0.89.

#### Pandemic-related stress

Pandemic-related life stress, job changes and financial difficulty was measured with a subset of items from the questionnaire of the Covid-19 and Perinatal Experiences Study (COPE Study, https://osf.io/uqhcv/). This questionnaire was designed to measure the impact of the COVID-19-related distress among mothers across countries and has been translated into eight languages. Participants reported on job changes and financial difficulty during the pandemic. More specifically, they indicated whether the following changes had occurred: loss of job, loss of hours, loss of health insurance, increased hours, increased responsibilities, increased monitoring and reporting, moved to remote working, decreased pay, decreased job security, reduced ability to afford childcare, reduced ability to afford rent/mortgage, having to fire or furlough employees, decrease in value of retirement, investments, or savings. Cronbach's alpha estimate of reliability was 0.83, which reveals these items could be summarized in a single index. Pandemic-related work stress was assessed by one item evaluating on a 1 (no distress) to 10 (severe distress) Likert scale the level of distress mothers experienced due to changes in work situation and financial difficulty caused by the COVID-19 pandemic. The correlation between job changes and work-related stress was *r* = 0.35, *p* < 0.001.

Pandemic-related life stress was measured with seven items of the COPE Study questionnaire: higher risk to COVID-19 due to existing medical condition(s), reduced access to foods or goods in the future, reduced access to medicine and hygiene supplies in the future, reduced access to baby supplies (e.g. formula, diapers, wipes) in the future, reduced access to mental health care in the future, reduced access to general health care in the future, reduced access to positive social interactions due to social distancing and/or quarantine. Items were rated on a Likert scale ranging from 1 (not of concern) to 4 (highly distressing). A total score was calculated by summing reported pandemic-related stress. Cronbach's alpha was 0.82.

#### Resilience

Resilience was measured with a 14-item scale (Wagnild & Young, 1993). Each item was answered using a 5-point Likert scale ranging from 1 (strongly disagree) to 5 (strongly agree), with total possible scores ranging from 14 to 70. Higher scores indicate higher levels of resilience. The RS-14 has been widely used in resilience research and has been translated into and validated in a variety of languages, such as simplified and traditional Chinese for mainland and Taiwanese Chinese participants, respectively (Chung et al., 2020; Tian & Hong, 2013). Cronbach's alpha was 0.85.

#### Family conflict

Family conflict was measured with six items of the COPE study questionnaire: verbally lashed out to your partner, verbally lashed out at your children, physically lashed out to my partner, physically lashed out to my children, less or not emotionally available to my partner, less or not emotionally available to my children. Parents indicated whether these situations happened in the past seven days on a scale ranging from 1 (not at all) to 5 (very often). A total score was calculated by summing the items. Cronbach's alpha was 0.85.

#### Demographic variables

According to the previous literature on mental health, the covariates included in the current study covered the following three domains: maternal characteristics, number of children, and age of the youngest child (Patrick et al., 2020; Prime et al., 2020). Maternal characteristics consisted of marital status (married, divorce and others), age (18–30, 30–35, 35–40, 40–60), educational level (college and below, university, postgraduate), employment (employed, unemployed), physical health (poor, fine, and good) and household income.

### Statistical analysis

Data analysis was performed using R software (version 4.0.2; R Core Team, 2020). Means and standard deviations were computed for continuous and normally distributed variables, and frequencies and percentages were used for categorical variables. One-way analyses of variance and chi-square tests were used to compare the differences between the three countries on continuous and categorical variables, respectively. The 27-item mental health scale was used as dependent variables in all cross-validation analyses. Ten-fold cross-validation models were applied to explore the predictive performance of related factors with maternal mental health, and also helpful to test how each country's best-performing model would perform predicting maternal mental health in the other countries. The R-package *xvalglms* (de Rooij & Weeda, 2020) allowed for conducting linear regression analyses using 10-fold cross-validation. Cross-validation allows for estimating how a model would perform on other samples. This out-of-sample predictive performance is more accurately determined by cross-validation than by traditional model fit measures such as *R*^2^ (Yarkoni & Westfall, 2017).

In this study, cross-validation analyses were conducted as the following: In the first step, 32 768 different regression models were specified by determining all possible combinations of the earlier identified 15 risk and protective factors. Next, the predictive performance of those models was evaluated separately for each country using 10-fold cross-validation. The method first divides the full country sample into 10 subsets, using one as training data, and nine of remaining subsets to validate the model estimated on the training process. Given that the characteristics of the training set are important in determining the predictive performance, the procedure of splitting the data in one training and nine validation sets is repeated 200 times for each model. Averaged across all repeats, the root mean square error of prediction (RMSEp) was used to evaluate the predictive performance of each model. Next, to determine the cross-cultural validity of the related factors correlated with maternal mental health in each country, the best-fitting model for one country was validated on the dataset of the other two countries. Finally, we conducted a robust standard OLS regression on each country's winning model to obtain the relative importance of the variables in each country in terms of standardized regression coefficients. In addition, following a suggestion of one of the reviewers, we conducted a post hoc exploratory analysis to examine whether resilience plays a moderating role in the relationship between maternal age, pandemic-related stress and maternal mental health.

## Results

Table 1 presents the descriptive analysis of the sample characteristics in China, Italy and the Netherlands during the COVID-19 pandemic. Almost all characteristics differed significantly across countries. In particular, socioeconomic (e.g. education, income, employment) and health (e.g. psychopathology, physical health conditions) variables differed between countries. In addition, there were large differences in grandparental support in childcare. Of all Chinese mothers, 53.6% indicated that one or more grandparents provided child care support, whereas only a small percentage in both the Netherlands (9.4%) and Italy (18.3%).

**Table 1.** Characteristics of Chinese, Italian and Dutch mothers/families during the COVID-19 pandemic

Characteristic	IT (*N* = 641)	NL (*N* = 900)	CH (*N* = 922)	*p* value	*Eta*^2^	*Cramer V*
Age mother				<0.001		0.154
18−30	49 (7.6%)	84 (9.3%)	133 (14.4%)			
30−35	148 (23.1%)	261 (29.0%)	325 (35.2%)			
35−40	231 (36.0%)	315 (35.0%)	348 (37.7%)			
40−60	213 (33.2%)	240 (26.7%)	116 (12.6%)			
Marital status				0.005		0.066
Married	612 (95.5%)	828 (92.0%)	876 (95.0%)			
Divorce and others	29 (4.5%)	72 (8.0%)	46 (5.0%)			
Education				<0.001		0.211
College and below	213 (33.2%)	308 (34.2%)	444 (48.2%)			
University	126 (19.7%)	365 (40.6%)	332 (36.0%)			
Postgraduate	302 (47.1%)	227 (25.2%)	146 (15.8%)			
Employment				<0.001		0.257
Employed	474 (73.9%)	645 (71.7%)	863 (93.6%)			
Unemployed	167 (26.1%)	255 (28.3%)	59 (6.4%)			
Household income (Euros/RMB: 10 000)				<0.001	0.062	
Mean (s.d.)	4.29 (1.81)	5.88 (2.40)	4.59 (3.39)			
Family conflict				<0.001	0.019	
Mean (s.d.)	2.01 (0.90)	1.69 (1.08)	1.95 (1.02)			
Number of children				<0.001		0.243
1	261 (40.7%)	232 (25.8%)	409 (44.4%)			
2	308 (48.0%)	440 (48.9%)	450 (48.8%)			
3 and more	72 (11.2%)	228 (25.3%)	63 (6.8%)			
Age youngest child				<0.001	0.090	
Mean (s.d.)	4.20 (2.72)	4.75 (2.94)	6.27 (2.67)			
Childcare during COVID-19 by grandparents				<0.001		0.442
No	534 (81.7%)	815 (90.6%)	428 (46.4%)			
Yes	117 (18.3%)	85 (9.4%)	494 (53.6%)			
Father involvement				<0.001	0.036	
Mean (s.d.)	2.23 (0.55)	2.30 (0.62)	2.51 (0.67)			
General psychopathology				<0.001	0.231	
Mean (s.d.)	2.03 (0.69)	1.52 (0.56)	1.26 (0.41)			
Pandemic-related work stress mother				<0.001	0.019	
Mean (s.d.)	6.89 (2.63)	4.58 (2.83)	4.75 (3.09)			
Pandemic-related life stress mother				<0.001	0.108	
Mean (s.d.)	2.41 (0.52)	1.94 (0.58)	2.20 (0.59)			
Resilience				<0.001	0.037	
Mean (s.d.)	52.37 (7.14)	55.71 (7.28)	56.07 (8.95)			
Physical health						
Poor	170 (26.5%)	176 (19.5%)	14 (1.5%)	<0.001		0.296
Fine	304 (47.4%)	445 (49.4%)	297 (32.2%)			
Good	167 (26.1%)	279 (31.0%)	611 (66.3%)			

### Cross-validation

Table 2 shows for each country the top three models identified through cross-validation in terms of minimizing the prediction error (RMSE) for the factors related to maternal mental health. Within countries, the top three models only slightly differed. Although there were some differences in the identified factors between countries, the results indicated that COVID-19-related stress, resilience, and marital conflict were important factors related to maternal mental health in each of the three countries.

**Table 2.** For each country, the factors related to maternal mental health according to the top three regression models identified through cross-validation

Independent variables	Italy			Netherlands			China		
Model ranking	1	2	3	1	2	3	1	2	3
Number of wins^a^	20%	15%	12%	49%	25%	9%	21%	19%	18%
Married							x	x	x
Number of children						x	x	x	x
Age youngest child									
Mother's age	x	x	x						
Mother's education				x	x	x	x	x	x
Family income		x	x				x	x	x
Mother's job				x	x	x		x	x
Pandemic-related life stress mother	x	x	x	x	x	x	x	x	x
Pandemic-related work stress mother	x	x	x	x	x	x	x	x	x
Resilience	x	x	x	x	x	x	x	x	x
Mother's poor physical health	x	x	x				x		x
Family conflict	x	x	x	x	x	x	x	x	x
Father's involvement	x	x	x						
Grandparents childcare							x	x	x
Grandparents childcare* Age of the youngest child		x			x				

a This indicates the percentage of the 200 cross-validation repeats a particular model showed the lowest prediction error (RMSE) of all 32 768 investigated models.

**Fig. 1.**
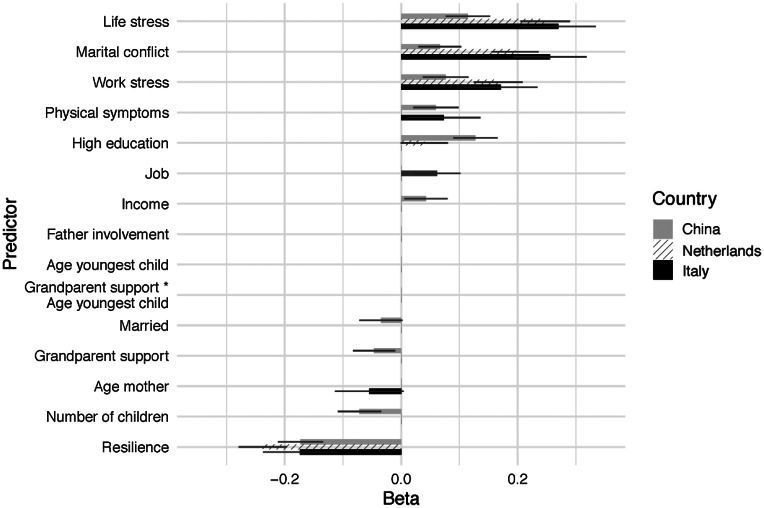
Differences between countries on continuous and dichotomous characteristics. To facilitate interpretation, coefficients of predictors not identified through cross-validation were fixed to zero.

Table 3 presents the results of the three regression analyses, for each country examining the best-performing model identified through cross-validation. In all countries, COVID-19-related stress, less resilience, and marital conflict showed a significant association with more mental health problems. In Italy, mothers' age was also associated with more mental health symptoms, as was maternal poor physical health. In the Netherlands, maternal high education and unemployment were positively related to mental health symptoms. In China, maternal high education, high family income, grandparental support, and physical health were all positively related to more mental health symptoms, while being married, and having more children were associated with less mental health symptoms. Figure 1 visualizes the differences among countries in the standardized regression coefficients of the predictors of maternal mental health. In addition, the post-hoc exploratory analysis showed that interactions between maternal age, pandemic-related stress, and resilience did not predict maternal mental health in Italy. However, resilience did buffer the negative effects of pandemic-related life/work stress on maternal mental health in the Netherlands, and seems to alleviate the impact of pandemic-related work stress on maternal mental health in China (see online Supplemental Materials: Table S1 and Fig. S1).

**Table 3.** The standardized regression coefficients (*β*) and Wald test *p* values according to robust regression analyses, including for each country only the best winning model

Variables	Italy		Netherlands		China	
	*β*	*p* value	*β*	*p* value	*β*	*p* value
Married					−0.089	0.003
Number of children					−0.114	<0.001
Age of youngest child						
Mother's age	−0.078	0.014				
Mother's education			0.060	0.022	0.175	<0.001
Family income					0.118	<0.001
Unemployment			0.075	0.004		
Pandemic-related life stress mother	0.288	<0.001	0.297	<0.001	0.158	<0.001
Pandemic-related work stress mother	0.184	<0.001	0.188	<0.001	0.109	<0.001
Resilience	−0.153	<0.001	−0.315	<0.001	−0.267	<0.001
Mother's poor physical health	0.100	0.003			0.063	0.046
Family conflict	0.255	<0.001	0.222	<0.001	0.148	<0.001
Father's involvement	0.056	0.086				
Grandparents childcare					−0.063	0.031
Grandparents childcare^a^ Age of the youngest						
*R*^2^	36.5%		41.9%		23.0%	

*Note*: Adjusted model *R*^2^ based on a linear ordinary least-squared regression model.

a Wald test *p* value <0.05.

**Fig. 2.**
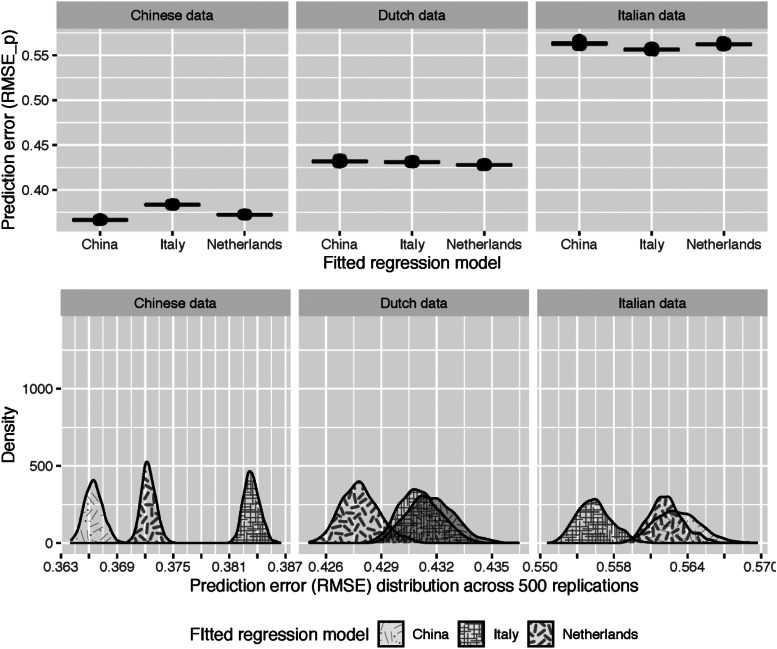
Boxplots (upper row) and density plots (bottom row) showing the distribution of the model prediction errors (RMSE) when fitting each country's best model to the dataset of each country.

Figure 2 visualizes the results from additional cross-validation analyses, which were conducted to evaluate the predictive performance of one country's best-performing model when predicting maternal mental health in the other two countries. As expected, the country's own top model showed the lowest prediction error for that country's data. However, there are overlapping distributions between the Dutch and the Italian model, which means that the factors associated with Dutch mothers' mental health are more similar to those related to the mental health of Italian mothers than to those of the Chinese mothers. In addition, we also found European models performed poorly in predicting maternal mental health in China.

## Discussion

In the current study, we examined risk and protective factors associated with maternal mental health during the COVID-19 lockdown in China, Italy, and the Netherlands. We applied a cross-validation approach (de Rooij & Weeda, 2020) for selecting the model with the best predictive performance in each of the three countries. Our results show that COVID-19-related stress and family conflict are shared risk factors and resilience is a shared protective factor in association with maternal mental health. In addition to these common factors, we identified unique best-performing models for each of the three countries. In Italy, mother's age and poor physical health were related to more mental health symptoms, while in the Netherlands, maternal high education and unemployment were associated with more mental health symptoms. In addition, high SES (mother's high education, high family income) and poor physical health were found to relate to a higher level of mental health symptoms among Chinese mothers. Moreover, having more than one child, being married, and grandparental support for mothers with a young child were important protective factors lowering the risk for mental health symptoms in China. Our findings indicate that, in addition to COVID-19-related stress, family conflict and resilience as shared factors, models examining maternal mental health during COVID-19 include distinct risk factors that are not replicated across cultures. Hence, although mothers with a young child globally suffer from mental health problems (Rahman, Surkan, Cayetano, Rwagatare, & Dickson, 2013), the constellation of factors associated with maternal mental health during COVID-19 is not identical across countries.

Our finding that pandemic-related work and life stress, family conflict, and resilience are shared factors related to maternal mental health problems in China, Italy, and the Netherlands is consistent with our hypothesis. Prime and colleagues found that family conflict, financial insecurities and social life disruptions due to COVID-19 contributed to worsen maternal mental health (Prime et al., 2020). Nevertheless, psychological resilience is vital for coping with adversity, uncertainty, and change (Killgore, Taylor, Cloonan, & Dailey, 2020). In accordance with earlier studies (Plomecka et al., 2020; Prime et al., 2020), the current study found that resilience can buffer the negative effects of pandemic-related life/work stress on maternal mental health in Netherlands, and moderates the impact of pandemic-related work stress on maternal mental health in China. However, interactions between maternal age, pandemic-related stress, and resilience did not predict maternal mental health in Italy.

Moreover, demographic characteristics, and physical health were associated with maternal mental health. Consistent with previous studies, we found that young, single, and unemployed mothers with poor physical health were prone to report more mental health symptoms (Liang, Berger, & Brand, 2019; Linares et al., 2020). More specifically, younger mothers usually have higher parenting stress than older mothers due to the low SES, and thus more likely develop depressive symptoms (Agnafors, Bladh, Svedin, & Sydsjö, 2019). Single mothers are at greater risk of worse mental health compared to mothers with a partner, because they tend to receive less social support (Liang et al., 2019). However, we suggest that due to the welfare policy and low levels of discrimination of single mothers in Italy and the Netherlands, marital status was not significantly associated with maternal mental health in the European countries (Pollmann-Schult, 2018).

Contrary to prior research reporting that higher levels of education and family income correlated with less mental health symptoms (Nisar et al., 2020), we found that highly educated mothers with high family incomes were more vulnerable during the pandemic. Disruptions in their support system may play a role: mothers with high family incomes might usually be able to rely on support from, e.g. housekeeping service and private kindergartens, but suddenly be confronted with increased burdens of housework and child care. It should be underscored that family income was only significant with maternal mental health in China. The association between education and worse maternal mental health was not significant in Italy, but only in the Netherlands and in China. One explanation for this pattern of findings could be that Italian gender inequality in child care persists also in highly educated families, even though high educational levels generally promote egalitarian gender roles (Dotti Sani & Quaranta, 2017). A previous cross-national comparison study conducted prior to COVID-19 showed that highly educated mothers in Italy often spend more time and energy on the child care than mothers in other European countries (Craig & Mullan, 2011) and receive low support from fathers.

Importantly, our cross-validation approach reveals between-country differences in other risk factors. One of the unique factors in the Chinese model was grandparental support, which significantly decreased maternal mental health symptoms in Chinese mothers, but not in Italian and Dutch mothers. As for the protective effect of grandparental support on Chinese mothers' mental health, grandparental childcare reduces mothers' parental burden and elevates their labor force participation, which in turn maximizes the wellbeing of the whole family (Croll, 2006). In this study, the grandparent effect was only observed in China, possibly because Chinese families have a high level of structural and functional solidarity. In fact, a high proportion of grandparents are co-resident with their grandchildren (Chen, Liu, & Mair, 2011) and therefore could provide childcare even during the lockdown. Indeed, our results showed that China's rate of grandparental childcare is more than twice compared to that of Italy and over five-fold of the Netherlands.

A second unique variable reflecting the cultural variation between the Netherlands and Italy *v.* China was the number of children. This study revealed that having more children related to better mental health for Chinese mothers, whereas this association was not found in Italian and Dutch mothers. China's one child policy has had a long-term effect on the family structure, and has led to smaller number of children in Chinese families. Compared to China, the Italian and Netherlands governments have provided social welfare for mothers and families with children (Knijn & Saraceno, 2010; Schleutker, 2014). Although previous research has demonstrated that two-child families in China have more parenting stress than one-child families (Hong & Liu, 2019), Chinese people tend to stick to the proverb ‘more children, more happiness’. Moreover, the crucial part of the traditional culture in China is family prosperity, implying that parents with more children would get more support from their children (Gao & Qu, 2019; Wu & Penning, 2019). Therefore, mothers with more children may be at lower risk of mental health symptoms.

Taken together, these results support our expectation that the factors related to maternal mental health differ across the three countries because of social and cultural variations. The distribution of the model prediction errors (RMSE) supports this claim. More specifically, the Italian model performed well in assessing the mental health of Dutch mothers and vice versa, probably because Italy and the Netherlands have more similar social welfare systems, living arrangements, and family compositions. However, risk and protective factors identified in these European countries' models perform poorly in predicting the maternal health of Chinese mothers.

### Limitation and implications

Several limitations should be noted. Firstly, maternal mental health was measured using a self-report scale that does not provide a clinical diagnosis. In addition, father involvement was measured with a new questionnaire, but items were comparable to previous studies on household and childcare tasks (de Meester, Zorlu, & Mulder, 2011), and the scale showed adequate internal consistency. Thirdly, there was not enough within-country variation in employment status to examine its relationship with maternal mental health in each country. Specifically, almost all mothers in China were employed. Consistent with our hypothesis, the Dutch unemployed mothers showed lower mental health, but this association was not found in Italy and China. Furthermore, the samples were recruited during different periods of the pandemic in the three countries. At the time, Italy was the most affected region by the COVID-19, followed by the Netherlands and China. Pandemic restrictions were, however, similar across countries during the period of data collection. Moreover, since the data were not representative of the populations of the three countries, we should be careful with generalizing the factors identified within a country to that country's entire population, because the factors may also differ between regions or subcultures within a country. Lastly, we recruited a convenience sample for which the nonresponse rate could not be established because of anonymity requirements. Thus, participants without internet connections (often living in poor rural areas) were underrepresented, which might have led to an underestimation of the mental health consequences of pandemic-related stress during the lockdown.

Despite these limitations, our study has several strengths compared to previous research. First, the cross-validation approach enables the identification of factors showing the best out-of-sample predictive performance. Indeed, the cross-validation approach avoids the risk of over fitting the regression model to the data by only fitting a single regression model on the entire dataset. By focusing on out-of-sample predictive performance, cross-validation models tend to have better reliability than standard regression models (Yarkoni & Westfall, 2017), which is essential given the difficulty of replicating the rare study setting of a pandemic. An additional strength of our study is reflected in the large samples collected from three countries. It enables us to examine the cultural variations of factors related to mental health among mothers from different countries during COVID-19 pandemic.

Finally, some important implications about maternal mental health across countries have been found. Firstly, it could help to identify features of vulnerable mothers in the three countries. Younger and less healthy mothers in Italy, and highly educated but unemployed mothers in the Netherlands may be at increased risk of poor mental health in times of pandemics. In China, without a spouse, mothers with less children, high SES, and poor physical health may be at higher risk of mental health symptomatology. Secondly, these results could provide effective advice in designing interventions that aim at focusing on improving maternal mental health in different national and cultural contexts. For instance, the current lockdown policy may ignore the advantages of grandparental childcare that support mother's mental health, as shown in China. Therefore, policy-makers could prioritize childcare support to families without grandparental support during lockdown. And the community-based organizations could create collaborative networks to provide programs and resources to support maternal mental health (Choi et al., 2020).

## Conclusion

This study used a cross-validation approach to examine the factors associated with maternal mental health during the COVID-19 lockdown in China, Italy, and the Netherlands. Our results showed that pandemic-related stress and family conflict are common risk factors and resilience is a shared protective factor related to maternal mental health across the three countries. Moreover, mothers' characteristics like age, education, physical health, employment and family income are significantly associated with mental health in the three countries. We also identified some distinct risk factors, such as grandparental support in childcare that were not replicated across cultures. These findings are important for the development of mental health promotion programs during COVID-19 and future pandemics.

## Supplementary material

For supplementary material accompanying this paper visit https://doi.org/10.1017/S0033291720005504. click here to view supplementary material
