# Supplementary material for: Maternal mental health during the COVID-19 lockdown in China, Italy, and the Netherlands: a cross-validation study
Source: Psychol Med. 2021 Jan 13:1–11. doi: 10.1017/S0033291720005504 (PMC7844185; doi:10.1017/S0033291720005504)
Supplement: Supplementary file 1 [file S0033291720005504sup.zip › S0033291720005504sup001.docx]

Table S1: The standardized regression coefficients (β) and Wald test p-values for the interaction effect of the resilience and pandemic-related stress according to robust regression analyses, including for each country only the best winning model.

| Variables | Italy | | Netherlands | | | China | |
| --- | --- | --- | --- | --- | --- | --- | --- |
|  | β | *p*-value | β | *p*-value | β | | *p*-value |
| Married |  |  |  |  | -0.091 | | 0.003 |
| Number of children |  |  |  |  | -0.114 | | <0.001 |
| Age youngest child | -0.079 | 0.021 |  |  |  | |  |
| Mother age |  |  |  |  |  | |  |
| Mother education |  |  | 0.061 | 0.018 | 0.173 | | <0.001 |
| Family income |  |  |  |  | 0.118 | | <0.001 |
| Unemployment |  |  | 0.082 | 0.001 |  | |  |
| Pandemic related life stress mother | 0.279 | <0.001 | 0.299 | <0.001 | 0.158 | | <0.001 |
| Pandemic related work stress mother | 0.195 | <0.001 | 0.200 | <0.001 | 0.120 | | <0.001 |
| Resilience | -0.167 | <0.001 | -0.353 | <0.001 | -0.271 | | <0.001 |
| Mother’s poor physical health | -0.101 | 0.003 |  |  | -0.059 | | 0.058 |
| Family conflict | 0.256 | <0.001 | 0.211 | <0.001 | 0.149 | | <0.001 |
| Father involvement | 0.051 | 0.119 |  |  |  | |  |
| Grandparents childcare |  |  |  |  | -0.063 | | 0.032 |
| Grandparents childcare *   Age youngest |  |  |  |  |  | |  |
| Mother age*Resilience | 0.001 | 0.969 |  |  |  | |  |
| Pandemic related life stress mother*Resilience | -0.030 | 0.463 | -0.139 | <0.001 | -0.011 | | 0.734 |
| Pandemic related work stress mother*Resilience | 0.049 | 0.252 | -0.056 | 0.041 | -0.070 | | 0.024 |
| R^2^ | 37.3% |  | 44.9% |  |  | |  |

Note: Adjusted model R^2^ based on a linear ordinary least squared regression model
* Wald test p-value < .05
