# Supplementary material for: Maternal mental health during the COVID-19 lockdown in China, Italy, and the Netherlands: a cross-validation study
Source: Psychol Med. 2021 Jan 13:1–11. doi: 10.1017/S0033291720005504 (PMC7844185; doi:10.1017/S0033291720005504)
Supplement: Supplementary file 1 [file S0033291720005504sup.zip › S0033291720005504sup002.docx]

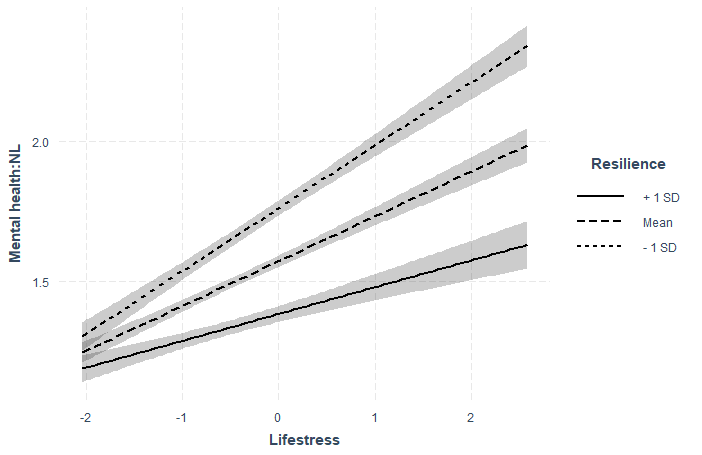

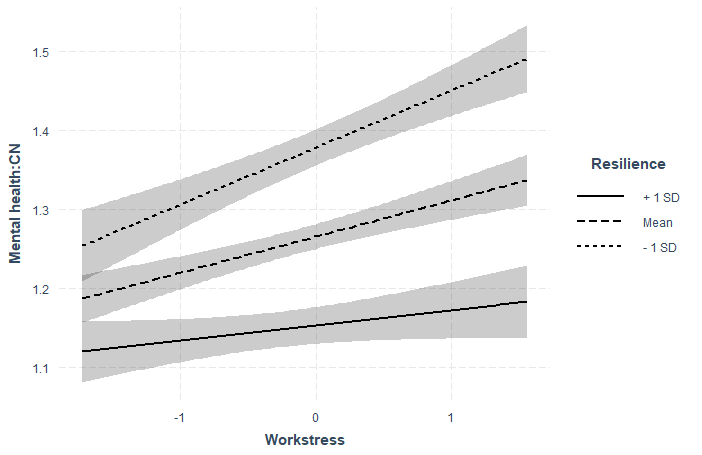


A：Resilience and life stress among Netherlands B：Resilience and work stress among Netherlands


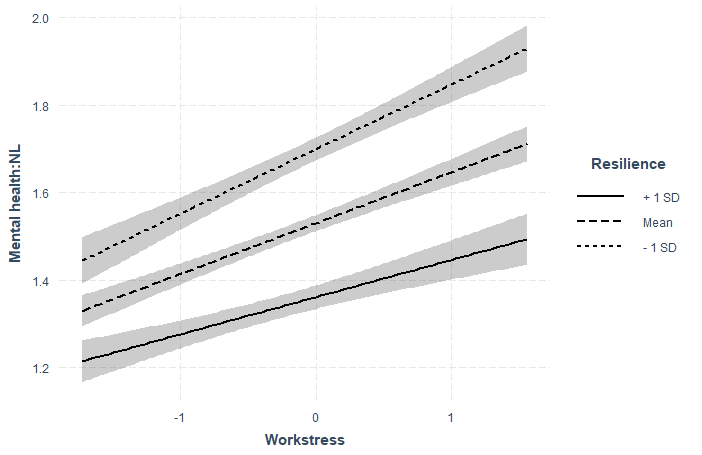

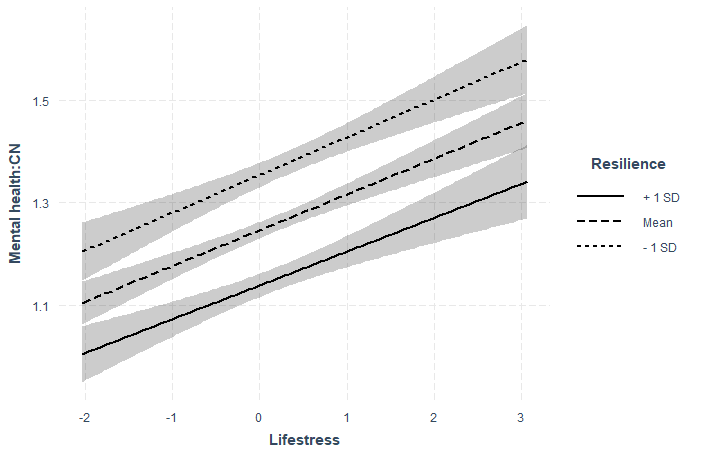


C：Resilience and life stress among China D：Resilience and work stress among China

Fig S1. The interaction of life stress, work stress, and resilience
